# Rhizospheric Bacteria From *Artocarpus heterophyllus* Exhibit Antimicrobial Traits and Antagonistic Activity Against *Pantoea stewartii* subsp. *stewartii*, Causal Agent of Jackfruit Bronzing Disease

**DOI:** 10.1155/ijm/6036078

**Published:** 2026-10-25

**Authors:** Jackeline Lizzeta Arvizu-Gómez, Alejandro Hernández-Morales, Christian González-Reyes, Cecilia Nheffreteri Cervantes-Díaz, Juan Campos-Guillén, Juan Ramiro Pacheco-Aguilar

**Affiliations:** ^1^ Plant-Microorganism Interaction Laboratory Office of Research and Graduate Studies Nayarit Center for Innovation and Technology Transfer (CENITT) Autonomous University of Nayarit Tepic Nayarit Mexico; ^2^ Faculty of Professional Studies Huasteca Zone Autonomous University of San Luis Potosi Ciudad Valles San Luis Potosi Mexico uaslp.mx; ^3^ Academic Unit of Chemical Biological and Pharmaceutical Sciences Autonomous University of Nayarit Tepic Nayarit Mexico; ^4^ Faculty of Chemistry Autonomous University of Queretaro Santiago de Queretaro Queretaro Mexico uaq.mx

**Keywords:** antagonistic activity, endophytes, jackfruit bronzing, rhizospheric bacteria, *P. stewartii subsp. stewartii*

## Abstract

The Jackfruit bronzing disease represents a significant phytosanitary constraint affecting *Artocarpus heterophyllus* production. In this context, the exploration of plant‐associated microorganisms as a source of potential biological control agents aligns with sustainable agricultural strategies. This study is aimed at isolating, characterizing, and evaluating the in vitro antagonistic interactions, and antimicrobial potential of indigenous rhizospheric and endophytic bacteria with the potential to be developed as biocontrol agents for the control jackfruit bronzing causal agent *Pantoea stewartii* subsp. *stewartii*. A total of 138 bacterial isolates were obtained from rhizospheric soils and internal plant tissues, with a predominance of rhizospheric bacteria (78.3%). In vitro antagonism assays revealed that only rhizospheric isolates exhibited inhibitory activity against the phytopathogen, identifying 12 strains with consistent antagonistic capacity. These isolates were further characterized to elucidate the potential mechanisms involved in microbial antagonism. Functional analyses demonstrated that the antagonistic isolates possessed multiple extracellular enzymatic activities, predominantly lipase, followed by amylase and cellulase activities, whereas protease activity was absent. Additionally, selected isolates exhibited biosurfactant production, suggesting their role in pathogen inhibition. Antibiosis was confirmed through the production of diffusible antimicrobial compounds. Furthermore, molecular screening revealed the presence of nonribosomal peptide synthetase (NRPS) gene fragments in several strains, supporting their genetic potential to synthesize bioactive secondary metabolites. Phylogenetic identification based on *16S rDNA* gene sequencing assigned the isolates to bacterial taxa known for their ecological versatility and functional attributes. Overall, this study provides evidence that rhizospheric bacteria associated with jackfruit harbor significant antagonistic and antimicrobial capabilities against jackfruit bronzing causal agent. These findings contribute to the understanding of microbial interactions and functional traits associated with plant microbiota. This study provides the first assessment of microorganisms associated with jackfruit.

## 1. Introduction

Jackfruit (*Artocarpus heterophyllus* Lam.), a tropical fruit tree belonging to the family Moraceae, is one of the crops with economic, social and cultural significance worldwide, mainly in producing countries. Currently, the jackfruit crop is cultivated in over 60 countries distributed throughout the South and Southeast Asian region, Oceania, the Caribbean, Latin America and East American, and parts of Africa, whose plantation areas and production have been reported to exceed 300,000 ha and 3.6 million tons of bulbs in the world. The economic value of jackfruit has gradually increased with planting in more areas [1–3]. According to latest research, the global jackfruit market was valued at USD 1.17 billion in 2025, reflecting steady growth. It is projected to expand at a compound annual growth rate (CAGR) of 6.3% from 2026 to 2035, reaching an estimated value of USD 2.07 billion by 2035 [4].

Nevertheless, the productivity of jackfruit has been threatened by pests and diseases. Among these, “jackfruit bronzing” disease is gaining relevance since it causes considerable losses in the Jackfruit industry. It has been estimated that the jackfruit bronzing disease causes damage with an average prevalence ranging between 20%–89%, with higher disease prevalence in the rainy season [5, 6]. Documented global distribution includes Southeast Asia, parts of East Asia, and Latin America [7–11]. Jackfruit bronzing disease is caused by the *Pantoea stewartii* subsp. *stewartii* phytopathogenic bacterium. This disease affects various parts of fruit such as pulp, rags, and skin. Typical symptoms of the disease are the presence of yellowish‐orange to reddish discoloration of the affected pulps and rags with the presence of bronzing specks all over the jackfruit pulps and rags [7, 12]. In general, jackfruit bronzing has been associated with internal symptoms without symptoms on the fruit surface. Recently, a work has reported the presence of external symptoms in field collected‐jackfruits infected by *P. stewartii* subsp. *stewartii*, such as abnormalities in the fruit stalk, fruit color, fruit shape, and spines [10].

Mexico is an important producer of jackfruit, with production highly concentrated in the state of Nayarit. According to official agricultural statistics, Mexico produced approximately 36,717 t of jackfruit in 2022, of which 34,844 t (94.9%) were produced in Nayarit, highlighting the importance of this crop to the regional agricultural economy. In Nayarit, jackfruit production is concentrated mainly in the municipalities of San Blas, Santiago Ixcuintla, and Compostela, which are therefore relevant areas for monitoring diseases affecting fruit quality. Jackfruit bronzing caused by *P. stewartii* subsp. *stewartii* was first reported in Mexico in 2017, following the detection of symptomatic fruits in major jackfruit‐producing areas of Nayarit. The current distribution and epidemiological status of jackfruit bronzing in Mexico require further systematic assessment [13, 14].

*P. stewartii*subsp.*stewartii* bacterium shows pathogenic variability for diverse hosts. In addition to jackfruit crop infection, this pathogen has the potential to infect different crops such as durian, longan, mango, pineapple, tomatoes, broccoli, pumpkin, cucumber, corn, maize, teosinte, rice, sweet potatoes, sugarcane, water spinach, peanuts, mustard and green beans [1, 10, 15]. The specific symptoms are dependent on the crop and plant′s susceptibility. Genetic diversity studies based on multilocus sequence analysis (MLSA) of housekeeping genes (*rpoB*, *gyrB*, *atpD*, and *infB*) showed the absence of genetic variations among *P. stewartii* subsp. *stewartii* isolates obtained from jackfruit crops of different regions [16]. Furthermore, phylogenetic analysis based in pathogenicity and virulence genes (*cpsD*, *cpsE*, and *hrp*) showed that *P. stewartii* subsp. *stewartii* isolates from jackfruit were clustered together with those obtained from maize [17]. Meanwhile, a comparative genome study of *P. stewartii*subsp.*stewartii* strains from corn versus jackfruit by using the RAST (rapid annotation using subsystem technology) server identified that 52% of genes fall into 27 active variants of subsystems [1].

Currently, the mitigation strategies of the jackfruit bronzing disease involve cultural practices and chemical control, the latter including bactericidal agents, insecticides and antibiotic treatments [1]. Biological control is considered a promising alternative to chemical control and has been globally increasing and gaining more attention because of its ability to control various phytopathogenic agents. In this context, biological control agents (BCAs), mainly bacteria, are among the most widely utilized, with a diverse range of bacterial genera having demonstrated potential as biocontrol agents for various plant diseases. Species within the genus *Bacillus*, *Alcaligenes*, *Arthrobacter*, *Azospirillum*, *Azotobacter*, *Pseudomonas*, *Rhizobium*, *Burkholderia*, *Klebsiella*, *Serratia*, *Enterobacter*, *Streptomyces*, *Paenibacillus*, and *Agrobacterium* have been identified with antagonistic potential against various phytopathogens, and some of these are already being marketed as plant protective products [18–20]. Most of successful BCAs correspond to microorganisms isolated from soil or in association with plants mainly from the endosphere, the phyllosphere or rhizosphere. Among these, the rhizobacteria and endophytic bacteria are the most widely reported with antagonistic potential against a wide range of plant pathogens [21]. Thus far, there have been very limited studies on the isolation and capability of BCAs with regard to jackfruit bronzing bacterium, *P. stewartii* subsp. *stewartii*. Only a recent work has reported the isolation of lactic acid bacteria (LAB) from fruit and vegetable supermarkets, others than jackfruit, with in vitro antagonistic potential against *P. stewartii* subsp. *stewartii* [22]. The search for BCAs based on the hosts or niches where they will be used could have advantages, since the microorganisms isolated may have adaptive capabilities to those niches, improving their effectiveness. Based on the above, this work aimed in the isolation and characterization of rhizobacteria and endophytic bacteria from jackfruit with antagonistic activities. Moreover, the ability to produce antimicrobial substances against jackfruit bronzing *P. stewartii* subsp. *stewartii* bacterium was in vitro tested.

## 2. Materials and Methods

### 2.1. Study Area and Collection of Plant Material and Soil Samples

Samples were collected from different jackfruit growing regions of the municipalities of San Blas, Compostela and Xalisco, Nayarit, Mexico (21°30’55.7”N 105°11’05.9”W, 21°27’20.9”N 105°11’13.3”W, 21°26’56.7”N 105°08’55.4”W, 21°25’28.0”N 105°11’06.6”W, 21°25’31.3”N 105°10’51.0”W, 21°24’37.5”N 105°10’37.0”W, 21°22’40.9”N 104°58’34.3”W, 21°10’33.8”N 105°10’12.8”W and 21°07’21.3”N 105°10’08.4”W) during September 2018. Branches from apparently healthy and undamaged plants of *A. heterophyllus* were collected and used as study material for the isolation of endophytic bacteria. For rhizosphere sampling, 10 soil samples were collected by adopting sampling methods previously described [23]. Samples of soil (500 g) were taken in proximity to the roots, after removing the surface layer along the roots to a depth of 20 cm. The soil close and attached to the roots was considered as rhizosphere. All samples were taken to the laboratory in sealed plastic bag and stored at 4°C.

### 2.2. Bacterial Isolation

Leaves of collected branches were firstly washed with tap water to remove dirt, followed by their transfer to a sodium hypochlorite solution (2%) for 2 min. The plant samples were subject to two washes with sterile distilled water for 2 min. From the final wash, 0.1 mL was poured into LB agar medium which was used as a control of the sterilization procedure. The surface sterilized plant material was cut into small fragments and crushed with sterile distilled water by using sterile mortar and pestle. A total of 2 mL of the macerate were inoculated in 25 mL of LB broth and incubated at 28 ± 2°C for 2 h with constant shaking. For the bacteria isolation from the rhizospheric soil, suspensions of each soil sample were prepared: 10 g of soil were added to 25 mL of LB broth and incubated at 28 ± 2°C with constant shaking for 2 h. From the suspensions (plant tissues and rhizosphere) serial dilutions up to 10^−6^ with saline solution (NaCl, 0.85%) sterile were performed. Aliquots (0.1 mL) of dilutions were plated on to LB agar plates and incubated at 28 ± 2°C for 48 h. Morphologically different bacterial colonies were subcultured and maintained in LB medium [24, 25].

### 2.3. In Vitro Antagonistic Activity

The antagonistic potential of the bacterial isolates of jackfruit against the *P. stewartii* subsp. *stewartii* phytopathogen bacterium was evaluated by dual culture assays using the pour plate diffusion method (PPD) and double agar layer (DLA) with some modifications [26, 27]. The *P. stewartii* subsp. *stewartii* JM‐5′ Mexican isolate, previously identified as jackfruit pathogen was used in these assays [8]. For the PPD assays, 0.1 mL from a culture of *P. stewartii* subsp. *stewartii* JM‐5 growth in LB broth for 24 h was mixed with LB agar, potato dextrose agar (PDA), or King B agar medium and poured onto the plates. The plates were allowed to solidify. The jackfruit bacterial isolates were seeded on these media by streak and the plates were incubated at 28 ± 2°C for 24–48 h, until the observation of growth inhibition zones around the streak. Alternatively, the jackfruit bacterial isolates were seeded on the media containing the *P. stewartii* subsp. *stewartii* JM‐5′ pathogen by stabbing using sterile toothpicks. The plates were incubated at 28 ± 2°C for 24–48 h. The presence of inhibition zones around the streak or stab was indicative of antagonistic activity. For the DLA method, an aliquot of the culture of each jackfruit bacterial isolate grown in LB medium was spotted in the center of a plate containing LB agar and then incubated at 28 ± 2°C for 48 h. Afterwards, a second layer of LB agar (5 mL) containing the *P. stewartii* subsp. *stewartii* JM‐5′ bacterium was added and incubated at 28 ± 2°C for 24–48 h. The appearance of clear zones around the bacterial spot was considered as positive for antagonism. All the tests were performed by triplicate. The jackfruit bacterial isolates with antagonist potential against *P. stewartii*subsp.*stewartii* JM‐5′ were selected and used in further experiments. Morphological characterization of the bacterial isolates was assessed involving culture properties like shape, color, elevation, edge, and texture of bacterial colony grown in LB media for 48 h. Furthermore, microscopic examination involved Gram staining and cell shape analysis, which were performed according to standard protocols (see Supporting Information 2).

### 2.4. Hydrolytic Enzymes Activity

The screening for extracellular hydrolytic activities (cellulase, amylase, protease, and lipase) was carried out by culturing the bacterial isolates onto specific media. All the hydrolytic enzyme assays were carried out in triplicate.

#### 2.4.1. Cellulase Activity

The production of cellulase enzymes qualitatively was tested by hydrolysis capacity [28, 29]. One loop of bacterial isolate was inoculated onto LB agar medium (1.5 g/L agar) supplemented with carboxymethylcellulase (CMC) (5 g/L). After incubation for 48 h at 28°C, the media were flooded with 0.5% (w/v) Congo red solution and kept for 15 min, then the plates were rinsed using 1 M NaCl. The formation of a clear zone around the colony was indicative of CMC hydrolysis capacity, which indicates synthesis of extracellular cellulase.

#### 2.4.2. Amylase Activity

The bacterial isolates were inoculated by stabbing onto LB agar medium (1.5 g/L agar) supplemented with 1% (w/v) starch and incubated at 28°C for 48 h. Afterwards, the revelation of the presence of clear zones around the colony, indicative of amylase activity, was carried out by adding iodine solution on plate surfaces [28].

#### 2.4.3. Protease Activity

Extracellular protease activity was detected by casein degradation as indicated by clearing zones on LB agar plates containing with 1% skim milk powder after 48 h of incubation at 28°C [30].

#### 2.4.4. Lipase Activity

The microorganisms were cultured on petri dishes containing medium supplemented with 1% (w/v) Tween 20 (peptone 10.0 g/L, NaCl 5.0 g/L, CaCl_2_.2H_2_O 0.1 g/L, agar 15 g/L, and pH 7.4) to verify the presence of halos formed by crystals, indicating lipase‐producing strains [31].

### 2.5. Biosurfactants Production Tests

The potential of jackfruit bacterial isolates to produce biosurfactants was assessed by the hemolytic activity assays and drop collapse test. The assays were performed for duplicate with two replicates.

#### 2.5.1. Hemolysis Test

The bacterial isolates were screened on blood agar plates containing 5% (v/v) blood and incubated at 28°C for 48 h. Hemolytic activity was detected as the presence of a clear zone around the colony. Hemolytic activity is considered by some authors as indicative of biosurfactant production.

#### 2.5.2. Drop Collapse Test

The assays were carried out based on the methodology previously reported with some modifications [32]. Briefly, each bacterial isolate was cultured in LB broth (10 mL) at 28 ± 2°C for 48 h with constant shaking. The supernatants of the cultures were recovered by centrifugation at 3000 rpm for 15 min. A total of 10 *μ*L of pure soybean oil were applied to a petri dish and allowed to equilibrate for 1 h. A total of 20 *μ*L of supernatant of the isolated to be evaluated was transferred to the oil drop, after 1 min the observation was carried out using a stereomicroscope (50X). A SDS 5% solution and LB broth were used as positive and negative control, respectively. The presence of biosurfactants was detected by the increase in the diameter of the drop or when the drop was flat.

### 2.6. Antimicrobial Activities

Production of antimicrobial compounds was evaluated by agar disk‐diffusion method with some modifications [33, 34]. The jackfruit bacterial isolate to be tested was inoculated in LB broth (5 mL), incubated at 28°C on rotary shaker at 200 rpm for 30 h. The bacterial cells were removed by centrifugation at 3500 rpm for 15 min and the supernatant was recovered. Sterile filter paper disks (6 mm) were soaked in the cell‐free supernatant (CSF) (100 *μ*L) and dried in a laminar flow hood. Suspension of *P. stewartii* subsp. *stewartii* JM‐5′ bacteria was spread on plates containing LB medium. Impregnated discs with supernatant were placed on LB medium inoculated with the pathogenic bacteria. The plates were incubated at 28°C for 48 h. The presence of growth inhibition haloes around disks was considered as indicative of the diffusible antimicrobial compounds synthesis. As controls, paper disks impregnated with LB broth and CFS obtained from a culture of the *Escherichia coli* TOP10 strain grown under similar conditions were used. The antimicrobial activity was estimated by measuring the diameter of the zone of inhibition formed around the disks with the aid of a ruler. Two independent experiments were conducted in duplicate. For data analysis, the diameter of the paper disk (6 mm) used in the assays was subtracted from the values obtained. The inhibition halo sizes are expressed as mean ± standard deviation (SD).

### 2.7. Amplification of Nonribosomal Peptide Synthetase (NRPS) Gene

Amplification of NRPS gene fragments coding for adenylation domains was conducted by PCR using degenerate primers 6R (5′‐SGAGTGSCCSCCSAGCTCGAA‐3′) and 5F (5′‐ATCGAGCTSGGSGAGATCGAG‐3′) [35]. Bacterial DNA was isolated using a protocol described previously [36]. The PCR conditions included an initial denaturation step at 94°C for 2.5 min; 35 cycles of denaturation at 94°C (30 s) followed by annealing at 55°C for 45 s, and extension at 72°C for 45 s; and a final extension step for 10 min at 72°C. Routine molecular techniques were performed using standard protocols [37]. DNA isolated from *Pseudomonas syringae* pv. *phaseolicola* NPS3121 and *E. coli* DH5 strains were used as positive and negative control, respectively.

### 2.8. Molecular Identification

Molecular identification of the bacterial isolates was performed by amplification, sequencing, and analysis of a *16S rDNA* gene region. The partial *16S rDNA* gene of each bacterial isolate was amplified from extracted DNA by PCR using universal primers FD1 and RD1 (5′‐ TCGTCGACAACAGAGTTGATCCTGGCTCAG‐3′, 5′‐ CCCGGGATCCAAGCTTAAGGAGGTGATCCAGCC‐3′, respectively) [38]. The PCR mixtures were denatured at 94°C for 2 min, which was followed by 35 cycles of 94°C for 30 s, 55°C for 45 s, and 72°C for 2 min and then a final extension at 72°C for 5 min. PCR products amplified with DNA polymerase high fidelity (Invitrogen, California, United States) were purified using the Gen Elute PCR Clean‐up kit (SIGMA, California, United States) according to the manufacturer′s instructions. The sequencing was performed at the Biotechnology Institute‐UNAM (IBT‐UAM, Morelos, Mexico). The obtained *16S rDNA* gene sequences were deposited in GenBank under the accession number: PZ449413, PZ449412, and PZ449414 for the isolate TLC‐2, TLC‐5, and TELLTB‐11, respectively. Bacteria were identified based on sequence similarities with homologous *16S rDNA* gene fragments using the BLASTn search algorithm. The closely related reference sequences‐based on BLAST results, adequate query coverage, sequence completeness‐ were retrieved from GenBank to determine the relation of our isolates with them. Phylogenetic analysis of nucleotide sequences obtained was performed by using ClustalW alignment software. The neighbor‐joining (NJ) method was used to cluster analysis using MEGA X software. The confidence of each branch was verified by bootstrapping 1000 times to construct phylogenetic tree [39–41].

## 3. Results and Discussion

### 3.1. Bacterial Isolates From Jackfruit Show In Vitro Antagonistic Activity Against Jackfruit Bronzing *P. stewartii* subsp. *stewartii* Bacterium

A total of 138 bacterial isolates with distinctive phenotypic characteristics among them were obtained from plant tissues (leaves) and rhizosphere of jackfruit crops. One hundred eight (78.3%) of these isolates were of rhizospheric origin, whereas 30 (21.7%) isolates corresponded to endophytic bacteria. The difference on microbial abundance in diverse regions or parts of plants such as endosphere and rhizosphere has been widely reported in several studies [42–45]. Typically, higher richness and diversity of the bacterial community are found in rhizosphere samples in relation to the endosphere. It has been postulated that the abundance and composition of the plant‐associated microbiota is based on enrichment and exclusion events of microbial communities. In this manner, enrichment processes driven by root exudation occur in the rhizosphere followed by exclusion events on the rhizoplane and within plants. This exclusion allows the selection of bacteria able compete in the presence of elevated nutrient levels which occur in the endosphere [46, 47].

In order to assess the antagonistic potential of the jackfruit bacterial isolates, in particular against *P. stewartii* subsp. *stewartii* JM5′ bacterium, in vitro confrontation assays were performed. The result of these assays demonstrated the capacity by some jackfruit bacterial isolates for growth inhibition of this phytopathogen. These antagonistic activities showed further to be dependent on the culture media used in the assays. Thus, 12 bacterial isolates (A‐5, A‐6, A‐8, A‐9, SJ‐4, SJ‐5, SJ‐6, SJ‐7, TLC‐2, TLC‐5, TLP4, and TELLTB‐11) showed clear growth inhibition zones of *P. stewartii* subsp. *stewartii* JM5′around of their colony or streak, supporting the antagonistic potential of these bacterial isolates (Figure 1) (see Supporting Information 1). These antagonistic activities were only observed in the confrontation assays performed on LB medium. No growth inhibitory effect was observed by the jackfruit bacterial isolates in assays using PDA or KB media. It is known that the synthesis of diverse secondary metabolites such as antimicrobial compounds depends not only on the microorganism but also on growth conditions (e.g. temperature or pH) and medium composition (carbon or nitrogen source). On the basis of the latter, the differences observed on the antagonistic capacity of the jackfruit bacterial isolates in function of the culture medium could be attributed to the medium composition. Several works have reported variability on antagonistic activities in some microorganisms when these are tested in different culture media, similar to that obtained in this study [48, 49].

**Figure 1:**
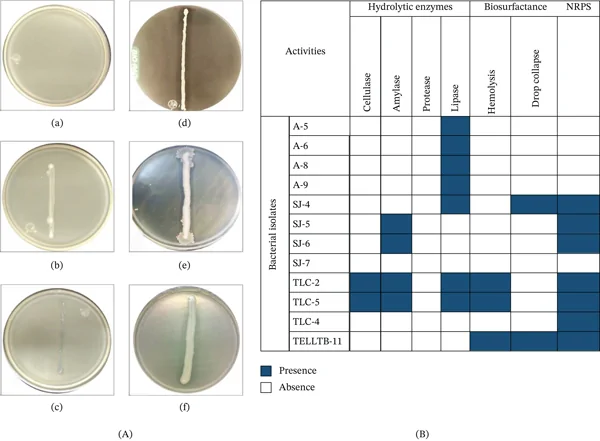
Antagonistic potential of bacterial isolates from jackfruit. Panel (A), representative pictures showing the antagonistic activity of (c) the TLC‐2, (d) TELLTB‐11, (e) TLC‐5, and (f) TLP‐4 isolates against *Pantoea stewartii* subsp. *stewartii* JM5′. Additionally, representative image of (b) confrontation assay using isolate that lacks inhibitory activity, and (a) control assay consisting of the *P. stewartii* subsp. *stewartii* JM5′ strain grown in the absence of any isolate is shown. Panel (B), profile of antagonistic activities of the bacterial isolates tested. All the tests were performed by triplicate.

All 12 bacterial isolates with in vitro antagonistic activity demonstrated through of these assays corresponded to rhizobacteria. Morphological characterization of the bacterial isolates was assessed (see Supporting Information 2). In general, these results highlight the biotechnological potential of jackfruit′s crops microbiota, in particular of rhizobacteria, demonstrating their antagonistic activity against jackfruit bronzing *P. stewartii* subsp. *stewartii* pathogen. To our knowledge, this is the first study that explores the jackfruit microbiota and demonstrates its antagonistic potential activity against pathogens of its host plant.

Similar to the findings of this study, previous works had also reported higher antagonistic activity against plant pathogens by rhizobacteria in relation to endophytic bacteria [50, 51]. The potential of rhizobacteria as BCA against different phytopathogens has been extensively demonstrated. They are potent microbial competitors mainly in the root zone. Through diverse mechanisms involving antibiosis, competition, induced systemic resistance of the host plant, hydrolytic enzymes, HCN production and/or siderophore production is how they carry out their antagonistic activity [19, 20]. The 12 bacterial isolates with antagonistic activity were selected for further experiments.

### 3.2. Hydrolytic and Biosurfactant Activities as Possible Mechanisms Involved in Antagonistic Potential of the Bacterial Isolates From Jackfruit

The capacity of hydrolytic enzymes production such as cellulase, amylase, protease, and lipase was evaluated for the 12 bacterial isolates. Among these, 16.7% possessed activities for three of the four evaluated enzymes, whereas 58% of the isolates possessed activities for only one enzyme. Protease activity was not observed in any of the isolates. The order of frequency of hydrolytic activities obtained among the isolates corresponded to lipase>amylase>cellulase (Figure 1).

Furthermore, because microbial biosurfactants have shown notable activity as antimicrobial agents [52], we evaluated the capacity of synthesis of biosurfactant compounds by the 12 bacterial isolates. Both biosurfactant screening tests performed (drop collapse test and hemolytic activity) showed the potential of some isolates for biosurfactant production (Figure 1). The TLC‐2, TLC‐5 and TELLTB‐11 isolates showed a clear zone around the colonies on the blood agar media, which is related with hemolytic activity. Although, the drop collapse tests showed that only SJ‐4 and TELLTB‐11 culture‐supernatants were able to produce biosurfactant compounds, which was evidenced by the formation of a flat drop during the assays reaching diameters of to 4 mm and 6 mm, respectively. Differences related to biosurfactant production ability by a bacterial isolate after evaluation using conventional tests such hemolysis and drop collapse test have been previously reported. Most of these reports have showed that hemolytic activity assays exhibit higher variability, mainly with a tendency toward negative results, in relation to those obtained through other biosurfactant screening methods [53, 54]. Even so, the hemolytic test, along with other tests (e.g. drop collapse test), remains part of the conventional tests used for the initial screening of biosurfactant. Reliable results correspond to those in which the synthesis profile is similar across several techniques. Based on this, the TELLTB‐11 isolate stands out because of its biosurfactant activity observed in both assays performed.

Microbial surfactants are complex molecules comprising different structures that include lipids, glycolipids, lipoproteins and/or polysaccharide‐protein complexes. Diverse biosurfactant like lipopeptides (surfactin, iturin, fengycin, etc.) and glycolipids exhibit strong antagonistic activity against pathogens. This antimicrobial activity arises from their ability to disrupt the lipid bilayer of membrane resulting cell lysis and/or by biofilm inhibition [52, 55].

On the other hand, the synthesis of extracellular enzymes (chitinase, cellulase, protease, amylase, and lipase) referred to as hydrolytic enzymes are among the main mechanisms used by diverse biocontrol bacteria having direct impact on phytopathogens by breaking down their cell walls or by disrupt the quorum sensing system of pathogens. Different groups of bacteria isolated from the rhizosphere, endosphere, and phyllosphere of different plant species have demonstrated their capacity of production of hydrolytic enzymes [56]. In general, these results confirm the antagonistic potential of jackfruit bacteria isolates which appear to be mediated by different mechanisms of control.

### 3.3. Antibiosis Mechanism by Jackfruit Bacterial Isolates

The involvement of antibiosis by the secretion of diffusible compounds in the antagonistic capacity of the 12 bacterial isolates was assessed in vitro. The CSF of the bacterial isolates were evaluated by disk diffusion method. The results of these assays confirm the antibacterial activity by all 12 bacterial isolates against the *P. stewartii* subsp. *stewartii* JM5′ bacterium which showed variable levels of activity (Figure 2). Among the evaluated isolates TLC‐5 and TELLTB‐11 exhibited the largest mean inhibition zones. These results confirm the antibacterial potential of the 12 bacterial isolates against the jackfruit‐bronzing pathogen bacterium. The production of diffusible compounds or antibiotics is another important inhibition mechanism that explains the efficacy of biocontrol bacteria. These microbial compounds encompass a chemically heterogeneous group of organic, low‐molecular‐weight compounds that negatively affect the growth or metabolic activities of other microorganisms [57]. Many biocontrol strains produce multiple antibiotics, which can suppress one or more pathogens [58]. Antibiotics are mainly peptides with low‐molecular weight generated ribosomally (e.g. bacteriocins) or nonribosomally (lipopeptides‐LPs, peptides, and polyketides) [59].

**Figure 2:**
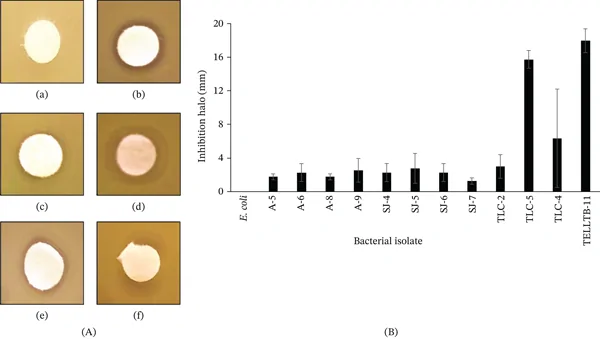
Antimicrobial activity of cell‐free supernatant (CFS) of the jackfruit bacterial isolates against *P. stewartii* subsp. *stewartii.* Panel (A), representative pictures showing the antimicrobial activity of (b) the A‐6, (c) SJ‐7, (d) TLC‐5, (e) TLC‐2, and (f) TELLTB‐11 isolates. (a) The CFS of the *E.coli* TOP 10 strain was used as control. Panel (B), bar graph showing the antimicrobial activity of the jackfruit bacterial isolates against *P. stewartii* subsp. *stewartii.* Each bar represent the mean of the diameter inhibition zone (mm) produced by the CFS of the jackfruit bacterial isolates. The values corresponding to the diameter of the filter paper used in these tests were subtracted from the reported values. The scatter bars represent SD, *n* = 4.

### 3.4. Antimicrobial Genetic Potential of Bacterial Isolates From Jackfruit

The genetic potential encoding enzymes involved in the synthesis of non‐ribosomal peptides (NRPs) was evaluated for the 12 isolates by PCR assays. NRPs are a broad class of compounds which are known for their antimicrobial properties through diverse mechanisms of action. Some well‐known antibiotics belong to this group of compounds. NRPs are synthesized by NRPSs which possess specific domains [60, 61]. PCR assays demonstrated the genetic capacity of NRPSs by some isolates tested. Amplicons of expected size (~450 bp) were obtained after the analysis of the SJ‐5, SJ‐6, TLC‐2, TLC‐5, TLP‐4, and TELLTB‐11 isolates (Figures 1 and 3). The remaining isolates did not show NRPSs genetic potential using this methodology. The general analysis of the assays performed showed that most of the isolates with NRPS coding capacity are those that also exhibited biosurfactant activity.

**Figure 3:**
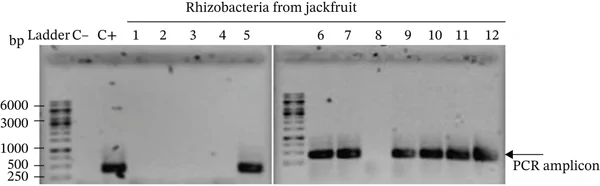
Genetic capacity for NRPs synthesis of the bacterial isolates from jackfruit. The presence of genes encoding peptide synthetase enzymes in bacterial isolates was evaluated using PCR assays with specific primers. Isolates with coding capacity exhibited an amplicon of approximately 450 bp. Negative control (C‐), *E. coli*; positive control (C+), *P. syringae* pv. *phaseolicola* NPS3121. (1) A‐5, (2) A‐6, (3) A‐8, (4) A‐9, (5) SJ‐4, (6) SJ‐5, (7) SJ‐6, (8) SJ‐7, (9) TLC‐2, (10) TLC‐5, (11) TLP‐4, and (12) TELLTB‐11. The GeneRuler 1 kb DNA Ladder (ThermoScientific) was used.

Overall, the results obtained provide evidence supporting the involvement of several complementary mechanisms as responsible of the antagonistic activity of the jackfruit bacterial isolates against *P. stewartii*subsp.*stewartii.* First, the detection of extracellular enzymes, which might contribute to antagonism by affecting cellular surfaces or processes associated with pathogen growth. Second, the biosurfactant assays indicating that some isolates, produced surface‐active compounds, which may contribute to antimicrobial activity through interactions with bacterial membranes. Third, the activity detected in CFS provides direct evidence that diffusible extracellular compounds are involved in the inhibition of *P. stewartii* subsp. *stewartii.* In addition, the detection of NRPS‐associated genetic determinants suggests a genetic potential for the production of nonribosomal antimicrobial metabolites. Nevertheless, the contribution of other mechanisms (e.g. competition for nutrients or volatile compounds) in the antagonistic activity of the selected isolates requiring further investigation.

Because the isolates TLC‐2 and TLC‐5 highlight for having the greatest number of properties related to biocontrol potential by exhibiting hydrolytic enzymes activity, biosurfactant and NRPS gene determinants, these isolates were selected for molecular identification purposes. Additionally, the TELLTB‐11 isolate was included because of its high antimicrobial activity, in addition to possessing biosurfactant activity and NRPS coding capacity.

### 3.5. Molecular Identification of Bacterial Isolates With Biocontrol Potential

The comparison of *16S rDNA* region sequences from the isolates TLC‐2 (PZ449413), TLC‐5 (PZ449412), and TELLTB‐11 (PZ449414) with those available in the GenBank database showed significant similarity of 99.7%–100%. Accordingly, the TLC‐2 and TELLTB‐11 isolates were identified as belonging to *Paenibacillus alvei* species whereas the TLC‐5 isolate was identified as *Acinetobacter bereziniae*. Phylogenetic analyses showed that TLC‐2 and TELLTB‐11 isolates clustered together in the *P. alvei* clade whereas TLC‐5 isolate showed similar sequence with *A. bereziniae* group, clustering in this clade (Figures 4 and 5). Phylogenetic trees validate the identity of the jackfruit bacterial isolates.

**Figure 4:**
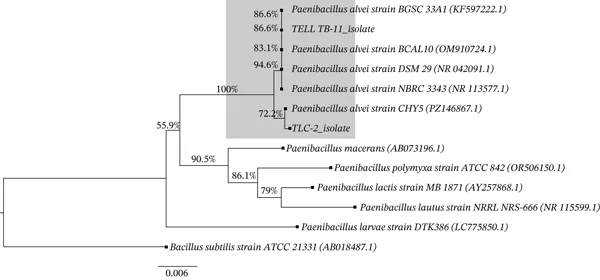
Phylogenetic tree of the *P. alvei* TLC‐2 and TELLTB‐11 isolates based on *16S rDNA* sequences analysis. MEGAX software was used to construct phylogenetic trees using neighbor joining (NJ) method. Bootstrap values was based on 1000 replicates. *B. subtilis* was used as outgroup.

**Figure 5:**
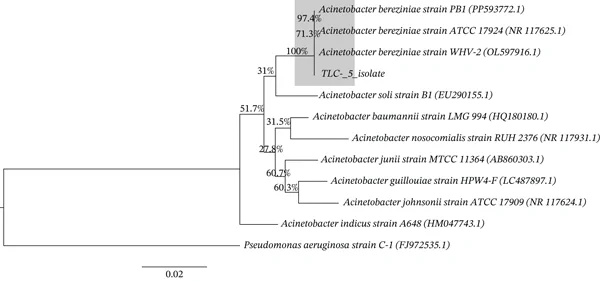
Phylogenetic tree of the *A. bereziniae* TLC‐5 isolate based on *16S rDNA* sequences analysis. MEGAX software was used to construct phylogenetic trees using neighbor joining (NJ) method. Bootstrap values was based on 1000 replicates *P. aeruginosa* was used as outgroup.

The isolation of strains of *A. bereziniae* and *P. alvei* from different habitats including endosphere and rhizosphere of various plant species have already been reported. Furthermore, properties related with antagonistic potential and plant growth promoting (PGP) traits have been reported for diverse isolates of these species [62, 63].

*A. bereziniae* is a predominantly environmental microorganism that has shown a rising frequency in clinical settings. Most of the documented works on *A. bereziniae* address the public health problems stemming mainly from the resistance to various antimicrobials that this microorganism presents. However, some researches have highlighted the potential of *A. bereziniae* as a PGPB through various mechanisms (IAA, siderophore, and exopolysaccharide production, P, Zn, and K solubilization, and ACC deaminase activity) [62, 64, 65]. Likewise, its antagonistic capacity against some phytopathogens, mainly fungal pathogens, has been demonstrated. So far, only the siderophore production and secretion of inhibitory molecules still unidentified have been suggested as some of the attributes that this species could present during its antagonism against phytopathogens [62, 63]. In the present study, it was demonstrated that *A. bereziniae* TLC‐5 isolate has multiple mechanisms of antagonistic activity which include glucanase, lipase, biosurfactance, and antibiosis. Further, NRPs antimicrobial genetic potential was demonstrated for this isolate thereby expanding the knowledge about arsenal of attributes that this species can possess. Nevertheless, given the clinical relevance of some *Acinetobacter* species, the biosafety of TLC‐5 must be carefully evaluated before considering its use as a BCA, particularly in crops intended for human consumption. Such assessments are essential to establish whether the beneficial traits of *A. bereziniae* TLC‐5 can be safely exploited while minimizing potential risk to human health and the environment. Therefore, the antagonistic activity and plant‐beneficial traits identified in TLC‐5 should not be interpreted as evidence of safety. In this regard, biosafety assessments of the TLC‐5 isolate are currently being conducted by our research group.

On the other hand, *P. alvei* has been isolated from a variety of environments but in majority this has been found in soil, in particular associated with plant roots. This species is recognized as a PGPB which exerts by diverse direct and indirect mechanisms. The most notable plant‐growth promoting feature of this *Paenibacillus* species is its variable biocontrol capabilities. *P. alvei* controls a diverse variety of phytopathogens by inducing the plant′s own resistance mechanisms (ISR) and/or producing biocidal substances [66–68]. The antimicrobials produced by this species include peptides (ribosomally synthesized and nonribosomally synthesized), hydrolytic enzymes, and volatile organic compounds (VOCs). Ribosomally synthesized and NRPs are among the main antimicrobials compounds of *P. alvei* [66–68]. In this work, differences on attributes related with antagonistic capacity were observed among the *P. alvei* TLC‐2 and TELLTB‐11 isolates. However, both isolates showed NRPS genetic potential. Despite, the *P. alvei* TLC‐2 isolate showed greater diversity of antagonistic attributes (hydrolytic enzymes, biosurfactant activity, and NRPS encoding capacity) in relation to *P. alvei* TELLTB‐11 isolate (biosurfactance and NRPS genetic potential), this latter exhibited a larger mean inhibition zone against *P. stewartii*subsp.*stewartii* demonstrated this by antibiosis assays. As above mentioned, antibiotics are primarily low‐molecular‐weight peptides produced through either ribosomal pathways, such as bacteriocins, or nonribosomal pathways, including lipopeptides, peptides, and polyketides. The differences of antibiosis potential observed among *P. alvei* TLC‐2 and TELLTB‐11 isolates could to be attributed to the chemical variability of antimicrobial compounds produced by these isolates. It is known that the antimicrobial activity of lipopeptides varies according to the lipid group length and carbon atoms content [68]. Likewise, differences in the potential for antimicrobial peptide synthesis among *P. alvei* isolates have been reported. *P. alvei* has the potential of produce a variety of antimicrobial peptides [69]. Compounds ranging from bacteriocin, lanthipeptide, sactipeptide, and lasso peptide are ribosomally‐synthesized, to a structural diversity of NRPs, polymyxin B, paenibacterin, paenibactin, anabaenopeptin NZ857, icosalide A/B, and bicornutinA1/A2, have been identified as potentially synthesizable by certain *P. alvei* isolates [69, 70]. In this study, by PCR assays was demonstrated the genetic capacity of NRPs in the *P. alvei* TLC‐2 and TELLTB‐11 isolates suggesting the potential of synthesis of some of these antimicrobials or even of new antimicrobials. More experimental work is still needed to demonstrate the expression of these gene determinants, the production of specific antimicrobial metabolites and establish their contribution to the inhibition of *P. stewartii*subsp.*stewartii.*

Notably, the isolates identified in this work exhibited diverse functional attributes that expand current knowledge regarding the antimicrobial and biotechnological potential of these taxa, particularly in the context of tropical fruit crop microbiomes.

As mentioned previously, there is only one report on the isolation of microorganisms, particularly LAB, with potential in vitro antagonistic activity against the causal agent of jackfruit bronzing, *P. stewartii* subsp. *stewartii* [22]. Therefore, the options for biocontrol microorganisms for implementation in integrated management strategies to control of jackfruit browning disease are still scarce. Similarly, only a few reports have been published about BCA microorganisms with potential against *P. stewartii* subsp. *stewartii* causes the Stewart′s wilt disease in corn crops. Endophytic fungi, endophytic actinobacteria and *Bacillus* isolates, all them obtained from Maize have been reported with capacity to suppress the growth of Stewart′s wilt *P. stewartii* subsp. *stewartii* pathogen [71–73].

From this study, bacterial isolates with antagonistic potential against *P. stewartii* subsp. *stewartii* pathogen causing of the emergence disease jackfruit bronzing were obtained contributing to repertoire of microbial options with view for its implementation in biocontrol strategies of this disease. Furthermore, the obtention of indigenous bacterial isolates could contribute to decrease the potential effects of these microorganisms on the micro‐ecosystem of the jackfruit, where these could be used.

Although the in vitro assays performed in this study provide valuable evidence of the antagonistic potential of the selected bacterial isolates against *P. stewartii* subsp. *stewartii*, these results should be interpreted within the experimental conditions under which they were obtained. The inhibition observed in culture‐based assays reflects the ability of the bacterial isolates to express antagonistic traits under controlled conditions and may not fully represent their performance in the complex environment of the plant. In particular, factors such as nutrient availability, temperature, pH, plant associated microbial communities, and host‐derive signals may influence the expression and effectiveness of antimicrobial compounds, hydrolytic enzymes, and other antagonistic mechanisms in planta. This consideration is consistent with the medium‐dependent antagonistic activity observed in the present study, in which growth inhibition was detected on LB medium but not on PDA or KB medium. Therefore, the in vitro findings should be regarded as an important first step for the selection and characterization of promising bacterial candidates rather than as direct evidence of biocontrol efficacy under field or plant‐associated conditions. Further in planta assays are warranted to determine the ability of the selected isolates to colonize plant tissues, suppress *P. stewartii* subsp. *stewartii* infection, and provide effective disease protection under biologically relevant conditions.

## 4. Conclusions

This study demonstrated that the jackfruit‐associated microbiota constitutes an important reservoir of bacteria with antagonistic and antimicrobial potential against *P. stewartii* subsp. *stewartii*, causal agent of jackfruit‐bronzing emergence disease. This is the first report related to jackfruit′s microbiota that assesses and demonstrate their potential use in the development of biotechnological processes. In this study, bacterial control agents against *P. stewartii*subsp.*stewartii* were successfully isolated, which showed a multifactorial arsenal of microbial traits associated with ecological fitness and antimicrobial activity. Molecular identification revealed that representative antagonistic isolates belong to the species *A. bereziniae* and *P. alvei.* This is the first study describing indigenous rhizospheric bacteria associated with *A. heterophyllus* with antagonistic activity against phytopathogens. These findings contribute to the understanding of microbial interactions within the jackfruit microbiome. Further studies will be necessary to validate their applicability as BCAs in integrated disease management programs.

## Funding

No funding was received for this manuscript.

## Conflicts of Interest

The authors declare no conflicts of interest.

## Supporting Information

Additional supporting information can be found online in the Supporting Information section.

## Supporting information


**Supporting Information 1** File S1: This word file contains images showing the antagonistic activity of the jackfruit bacterial isolates against *P. stewartii* subsp. *stewartii* by in vitro confrontation assays using LB medium.

**Supporting Information 2** File S2: This word file contains a table listing the characteristics of the colony and cell morphologies of the jackfruit bacterial isolates with in vitro antagonistic activity.

## Data Availability

The data that support the findings of this study are available on request from the corresponding author.
