# Supplementary material for: Rhizospheric Bacteria From Artocarpus heterophyllus Exhibit Antimicrobial Traits and Antagonistic Activity Against Pantoea stewartii subsp. stewartii, Causal Agent of Jackfruit Bronzing Disease
Source: Int J Microbiol. 2026 Oct 25;2026:6036078. doi: 10.1155/ijm/6036078 (PMC13695870; doi:10.1155/ijm/6036078)
Supplement: Supplementary file 1 [file IJM-2026-6036078-s001.docx]

**Supplementary material.**

**Supplementary material 1.** Macroscopic and microscopic morphology of Jackfruit rhizobacteria with potential in vitro antagonistic activity.

|  | **Colony Morphology** | | | | | | | | **Cell Morphology** | | |
| --- | --- | --- | --- | --- | --- | --- | --- | --- | --- | --- | --- |
| **Isolate** |  | **Form** | **Margin** | **Elevation** | | **Surface** | **Texture** | **Colour** |  | **Shape** | **Gram staining** |
| A-5 |  | round | undulate | convex | | smooth | dry | white |  | cocci | negative |
| A-6 |  | round | undulate | convex | smooth | | dry | white |  | cocci | negative |
| A-8 |  | round | undulate | convex | | smooth | dry | white |  | cocci | negative |
| A-9 |  | round | undulate | convex | | Smooth | dry | white |  | cocci | negative |
| SJ-4 |  | round | undulate | convex | | smooth | dry | white |  | cocci | negative |
| SJ-5 |  | round | undulate | convex | | smooth | dry | yellow-green |  | cocobacilli | negative |
| SJ-6 |  | punctiform | entire | convex | | smooth | dry | white |  | bacilli | negative |
| SJ-7 |  | round | entire | convex | | smooth | viscid | white- translucent |  | bacilli | negative |
| TLC-2 |  | irregular | entire | convex | | smooth | dry | white |  | bacilli | negative |
| TLC-5 |  | irregular | entire | convex | | smooth | dry | white |  | cocobacilli | negative |
| TLP-4 |  | round | irregular | convex | | smooth | mucoid | yellow-green |  | bacilli | negative |
| TELLTB-11 |  | irregular | irregular | convex | | rough | dry | white |  | bacilli | negative |

Bacterial morphology on LB medium
