# Supplementary material for: Rhizospheric Bacteria From Artocarpus heterophyllus Exhibit Antimicrobial Traits and Antagonistic Activity Against Pantoea stewartii subsp. stewartii, Causal Agent of Jackfruit Bronzing Disease
Source: Int J Microbiol. 2026 Oct 25;2026:6036078. doi: 10.1155/ijm/6036078 (PMC13695870; doi:10.1155/ijm/6036078)
Supplement: Supplementary file 2 [file IJM-2026-6036078-s002.docx]

**Supplementary material.**

**Supplementary material 1.** In vitro antagonistic activity of the Jackfruit-bacterial isolates against Pantoea stewartii subsp. stewartii

| a-5   | a-6   | a-8   | a-9   |
| --- | --- | --- | --- |
|  |  |  |  |
| SJ-4   | **SJ-5**   | **SJ-6**   | **SJ-7**   |
|  |  |  |  |
| TLP-4   | **TLC-2**   | **TLC-5**   | **TELLTB-11**   |
|  |  |  |  |
